# Dietary administration of the probiotic *Shewanella putrefaciens* to experimentally wounded gilthead seabream (*Sparus aurata* L.) facilitates the skin wound healing

**DOI:** 10.1038/s41598-020-68024-z

**Published:** 2020-07-03

**Authors:** Zhichu Chen, Diana Ceballos-Francisco, Francisco A. Guardiola, M. Ángeles Esteban

**Affiliations:** Fish Innate Immune System Group, Department of Cell Biology and Histology, Faculty of Biology, Regional Campus of International Excellence, Campus Mare Nostrum, University of Murcia, 30100 Murcia, Spain

**Keywords:** Biological models, Animal disease models

## Abstract

The effect of the probiotic *Shewanella putrefaciens* Pdp11 (SpPdp11) was studied on the skin healing of experimentally wounded gilthead seabream (*Sparus aurata* L.). Two replicates (n = 12) of fish were fed CON diet or SP diet for 30 days. Half of the fish were sampled while the others were injured and sampled 7 days post-wounding. Results by image analysis of wound areas showed that SpPdp11 inclusion facilitated wound closure. Compared with the CON group, fish in SP group sampled 7 days post-wounding had a significantly decreased serum AST and increased ALB/GLOB ratio. Furthermore, protease and peroxidase activities were significantly increased in skin mucus from fish in SP group sampled 7 days post-wounding, compared with those fed CON diet. Additionally, SP diet up-regulated the gene expression of antioxidant enzymes, anti-inflammatory cytokines, and re-epithelialization related genes in the fish skin. Furthermore, significant decreases in pro-inflammatory cytokines expression were detected in fish from SP group, respect to control ones. Overall, SpPdp11 inclusion facilitated the wound healing and the re-epithelialization of the damaged skin, alleviated the inflammatory response in the wound area through intensifying the antioxidant system, and enhancing the neo-vascularization and the synthesis of matrix proteins in the skin wound sites of fish.

## Introduction

The vertebrate integument (skin) is an organ consisting of the epidermis, dermis, and hypodermis. The skin is the prominent mucosal surface for crucial interface contact and communication with its external milieu^1, 2^. The presence of a layer of external mucus in the skin is one of the most distinctive features in teleost. Furthermore, the skin mucus is in direct contact with all the substances and microorganisms present in the aquatic environment^3^. The mucous secretory cells, macrophages, and various lymphocytes ubiquitous in the skin and formulate an intact barrier function, which maintains the homeostasis of fish^4^. However, in nowadays, high density and intensification of re-circulating aquaculture systems facilitate the skin lesions, abrasions and ulcers in farmed fish, which allow the colonization by commensal (typically with low pathogenicity) and opportunist pathogenic microorganisms^5^. Rapidly-repaired skin makes a case for the defensive mechanism against the external environment^6,7^. It is considered of great interest to find new measures for facilitating wound healing, which will improve the wellbeing and health of farmed fish.

The phases of teleost wound healing involve inflammation, re-epithelialization, and new tissue formation and remodeling, except the blood clot formation posed in mammalian^7–9^. In mammalian, the inflammatory phase, ascribes local release of pro-inflammatory cytokines and subsequent recruitment of neutrophils and macrophages, is related to remove cellular debris and pathogens from host tissue, as well as the release of cytokines and growth factors for initiating the next phase of the healing process^10,11^. Nevertheless, studies on adult zebrafish indicated that re-epithelialization is independent of inflammation in fish^8^, it has also been elucidated that the re-epithelialization in scale removed seabream also happened before the activation of leucocytes and induction of macrophages^7^. By comparison, fibroblast recruitment, granulation tissue formation, and wound vascularization seem presumably attributed to the inflammation both in mammals and fish^8,12^. To provide the experimental basis and theoretical basis for screening nutritional therapies that promote fish wound healing, it will help to elucidate the specific effects of additives on each stage of the wound healing. Till present, few studies have focused on how nutrition or some functional foods can improve the skin wound healing on fish^13–15^.

Many studies indicated that probiotics, which were defined as live microbial feed supplements that protect the farmed fish from stress and infection under intensive culture conditions, could replacing conventional drugs^16–18^. *Shewanella putrefaciens* Pdp11 (known as SpPdp11), which is a gram-negative pleomorphic bacterium that was isolated from the skin of healthy specimens of gilthead seabream (*Sparus aurata L.*), has already been verified as a probiotic for several farmed marine fish species, including larvae and juveniles specimens^19–26^. Homing in on the skin immune-related studies, the results on gilthead seabream indicated that dietary administration of SpPdp11 could increase the skin mucosal immunity, as well as up-regulated gene expression in the skin of different immune-related genes, leading a more robust skin barrier which could be correlated with higher protection against stressors or diseases^19^. In an in vitro study, it was demonstrated that SpPdp11 was able to ameliorate the negative effects caused in the ventral skin of gilthead seabream by the pathogen *Photobacterium damselae* subsp. *piscicida,* by studying the expression of several cytokines^21^. Taken into account all these considerations and our previous studies^20,22^, the aim of the present work is to study the effect of the dietary administration of SpPdp11 on the skin wound healing of experimentally wounded gilthead seabream (*S. aurata*) specimens. The results will contribute to the manipulation of fish diet to reinforce fish immunity and to improve the innate defenses, helping to reduce the use of antibiotics or other chemical products and promoting aquaculture in a more environmentally sustainable way. Gilthead seabream has been selected as a representative species of the Mediterranean aquaculture.

## Results

After 30 days of feeding the fish with the experimental diets (CON or SP), four specimens per tank were sampled while the other four specimens were wounded immediately and sampled 7 days post-wounding.

### Image analysis of wound areas

**Figure 1:**
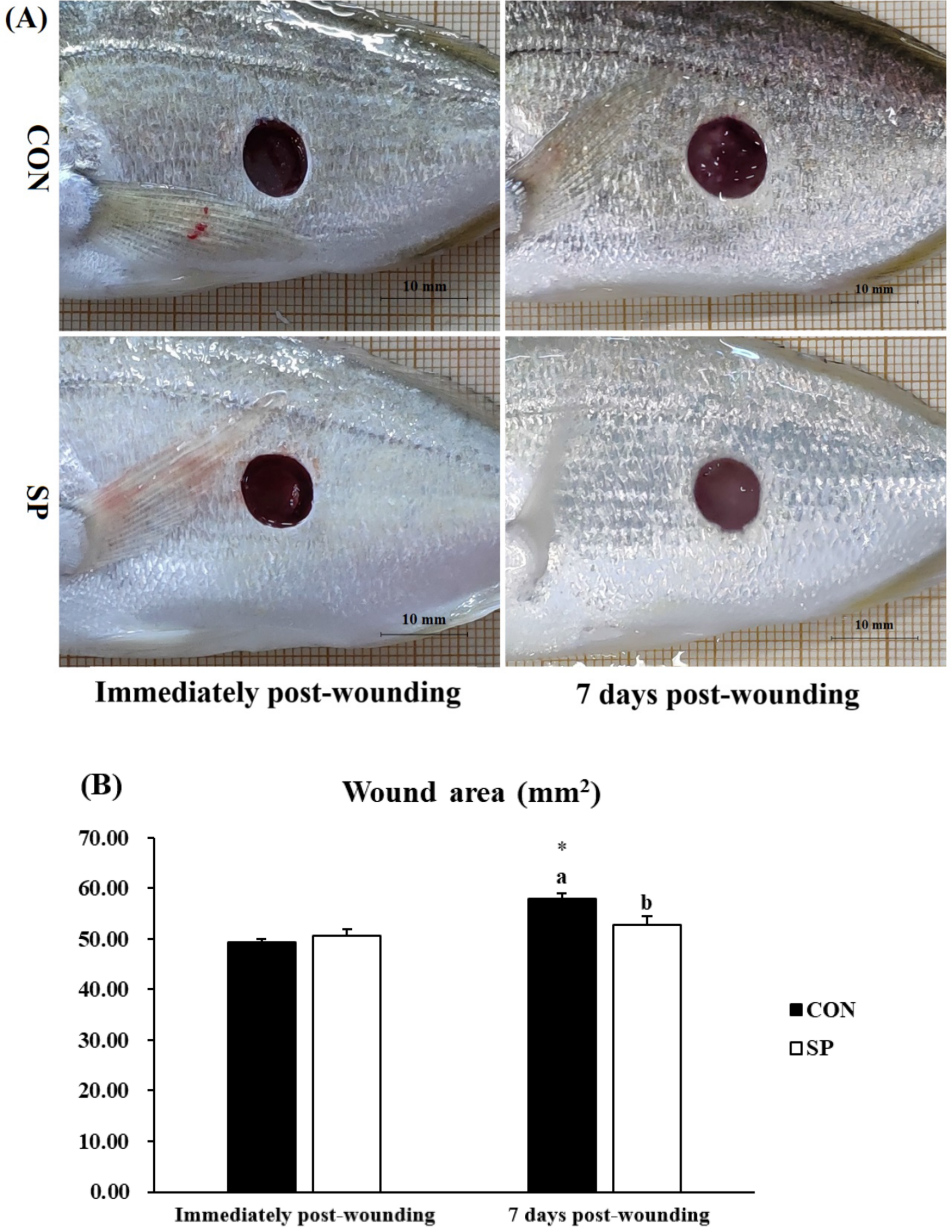
Experimental wounds below of the lateral line on the left flank of gilthead seabream specimens. Time-lapsed images (**A**) and wound area in mm^2^ (**B**) on gilthead seabream fed CON (control, black bars) and SP (control diet enriched with 10^9^ cfu g^−1^ SpPdp11, white bars) diet sampled pre-wounding or 7 days post-wounding. The wound area in mm^2^ were quantified using IMAGE-PRO PLUS Version 6. Error bars of columns denote standard error of means (n = 8). Different letters denoted significant differences between diets and asterisks indicated differences between time points (*P* < 0.05).

After being injured, all the wounds were photographed and new photographs of them were taken 7 days post-wounding to study the wound aspect and the healing progress in both groups of fish (fed CON or SP diet) (Fig. 1A). The wound areas of all the experimental fish were similar (*P* > 0.05) when determined immediately after wounding.

There was a significant (*P* < 0.05) increment between the wound areas of CON group pre-wounding and CON group 7 days post-wounding, but not the same for the SP group (*P* > 0.05) (Fig. 1B). There is a significantly (*P* < 0.05) reduced wound area in the SP group by comparing it with the CON group 7 days post-wounding by quantification (Fig. 1B).

### Serum metabolic parameters

The results obtained for the serum metabolic parameters studied on gilthead seabream fed CON and SP diets at the two sampling times (Pre-wounding and 7 days post-wounding) are shown in Table 1. Pre-wounding fish in the SP group had no significant (*P* > 0.05) differences in any of the studied serum parameters compared with the CON group. In the CON group, the skin wounds significantly (*P* < 0.05) enhanced the serum AST, UA, GLU, and PHOS concentrations. On the contrary, the skind wounds significantly (*P* < 0.05) reduced ALB/GLOB ratio, while no significant (*P* > 0.05) changes were observed in any other studied serum parameters. However, in the SP group, the serum UA, GLU, and PHOS concentration 7 days post-wounding were significantly (*P* < 0.05) higher, while the other parameters were not significantly (*P* > 0.05) changed compared with the data obtained for pre-wounding fish. Only statistically significant differences (*P* < 0.05) were recorded in the serum AST concentration (which was decreased) and ALB/GLOB (which was increased) in the SP group compared with CON group 7 days post-wounding, while the other parameters remained not significantly (*P* > 0.05) changed.

**Table 1:** Metabolic parameters in the serum of fish fed CON (control) and SP (control diet enriched with 10^9^ cfu g^−1^ SpPdp11) diet sampled pre-wounding or 7 days post-wounding.

Parameters	Pre-wounding		7 days post-wounding	
	CON	SP	CON	SP
AST (U/ml)	36.60 ± 2.20	33.5 ± 12.50	44.33 ± 5.61^a^*	23.00 ± 2.08^b^
CK (U/ml)	2,830.00 ± 35.00	3,088.83 ± 532.58	2,914.75 ± 300.19	2,475 ± 737.00
UA (mg/dL)	0.13 ± 0.025	0.13 ± 0.03	0.58 ± 0.14*	0.55 ± 0.05*
GLU (mg/dL)	106.60 ± 11.99	83.50 ± 9.92	180.25 ± 20.70*	202.00 ± 10.27*
Ca^2+^ (mg/dL)	12.70 ± 0.38	12.22 ± 0.23	13.90 ± 0.49	13.42 ± 0.16
PHOS (mg/dL)	9.34 ± 0.40	9.04 ± 0.49	12.03 ± 0.70*	12.14 ± 0.56*
ALB/GLOB	1.13 ± 0.12	1.15 ± 0.04	0.94 ± 0.06^b^*	1.20 ± 0.08^a^
K^+^ (mM)	7.60 ± 0.20	7.48 ± 0.19	7.16 ± 0.32	7.46 ± 0.28
Na^+^ (mM)	165.40 ± 1.17	168.20 ± 0.80	173.50 ± 1.32	173.50 ± 1.66

Data represent the mean ± SEM (n = 4). Different letters denoted significant differences between diets and asterisks indicated differences between time points (*P* < 0.05).

*AST* aspartate aminotransferase, *CK* creatine kinase, *UA* uric acid, *GLU* glucose, *Ca*^*2*+^ calcium, *PHOS* phosphorus, *ALB/GLOB* albumin/globulin ratio, *K*^+^ potassium, *Na*^+^ sodium.

### Skin mucus immune parameters

**Figure 2:**
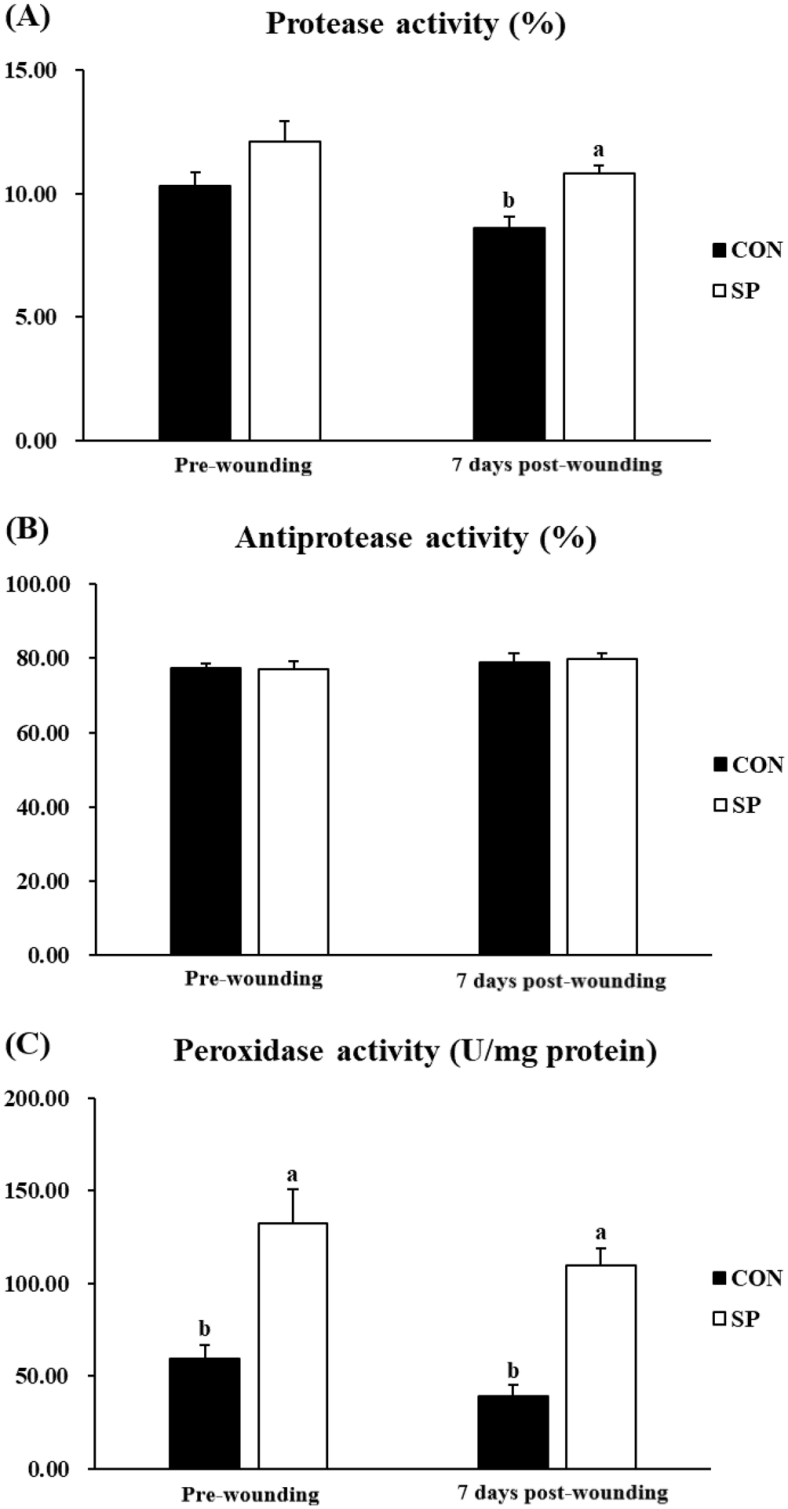
(**A**) Protease (%), (**B**) anti-protease (%), and (**C**) peroxidase (U/mg protein) activities in the skin mucus of gilthead seabream fed CON (control, black bars) or SP (control diet enriched with 10^9^ cfu g^−1^ SpPdp11, white bars) diet sampled pre-wounding or 7 days post-wounding. Error bars of columns denote standard error of means (n = 8). Different letters denoted significant differences between diets (*P* < 0.05).

The results obtained for the skin mucus immune parameters are presented in Fig. 2. There were not significant (*P* > 0.05) differences in protease, anti-protease, and peroxidase activities between pre-wounding and 7-days post-wounding fish in both CON group and SP group (Fig. 2). Pre-wounding fish in the SP group showed a statistically significant (*P* < 0.05) enhanced peroxidase activity and a similar (*P* > 0.05) protease and anti-protease activities compared with the fish in CON group pre-wounding (Fig. 2). Fish sampled 7 days post-wounding, showed significant (*P* < 0.05) enhanced protease and peroxidase activities although no significant (*P* > 0.05) differences were recorded in anti-protease activity from fish in the SP group compared with those of the CON group (Fig. 2).

### Gene expression profile in skin

**Figure 3:**
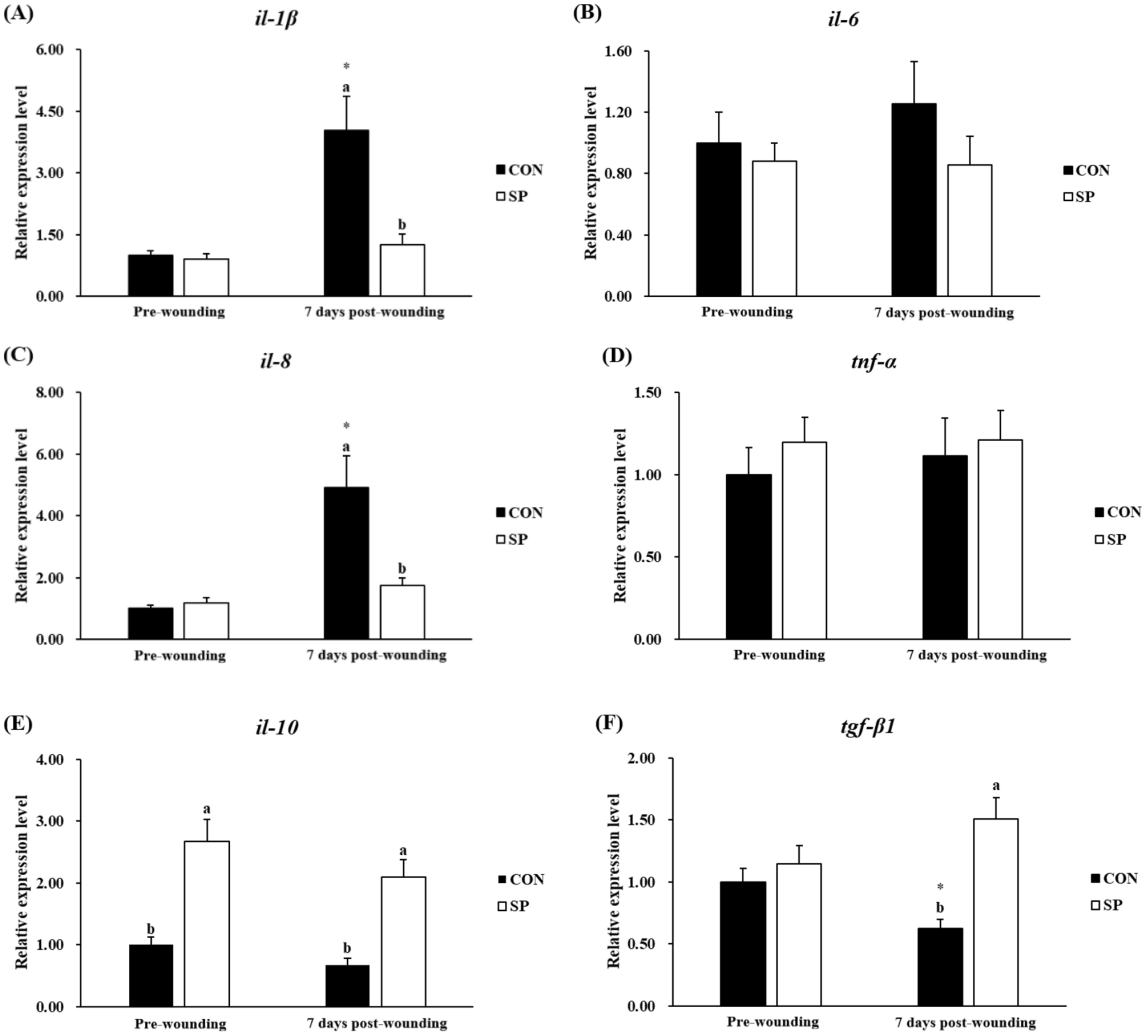
Relative expression level of pro-inflammatory (*il-1β*, *il-6*, *il-8*, and *tnf-α*), and anti-inflammatory cytokines (*il-10* and *tgf-β*) genes in skin samples of gilthead seabream fed CON (control, black bars) or SP (control diet enriched with 10^9^ cfu g^−1^ SpPdp11, white bars) diet sampled pre-wounding or 7 days post-wounding. Error bars of columns denote standard error of means (n = 8). Different letters denoted significant differences between diets and asterisks indicated differences between time points (*P* < 0.05).

**Figure 4:**
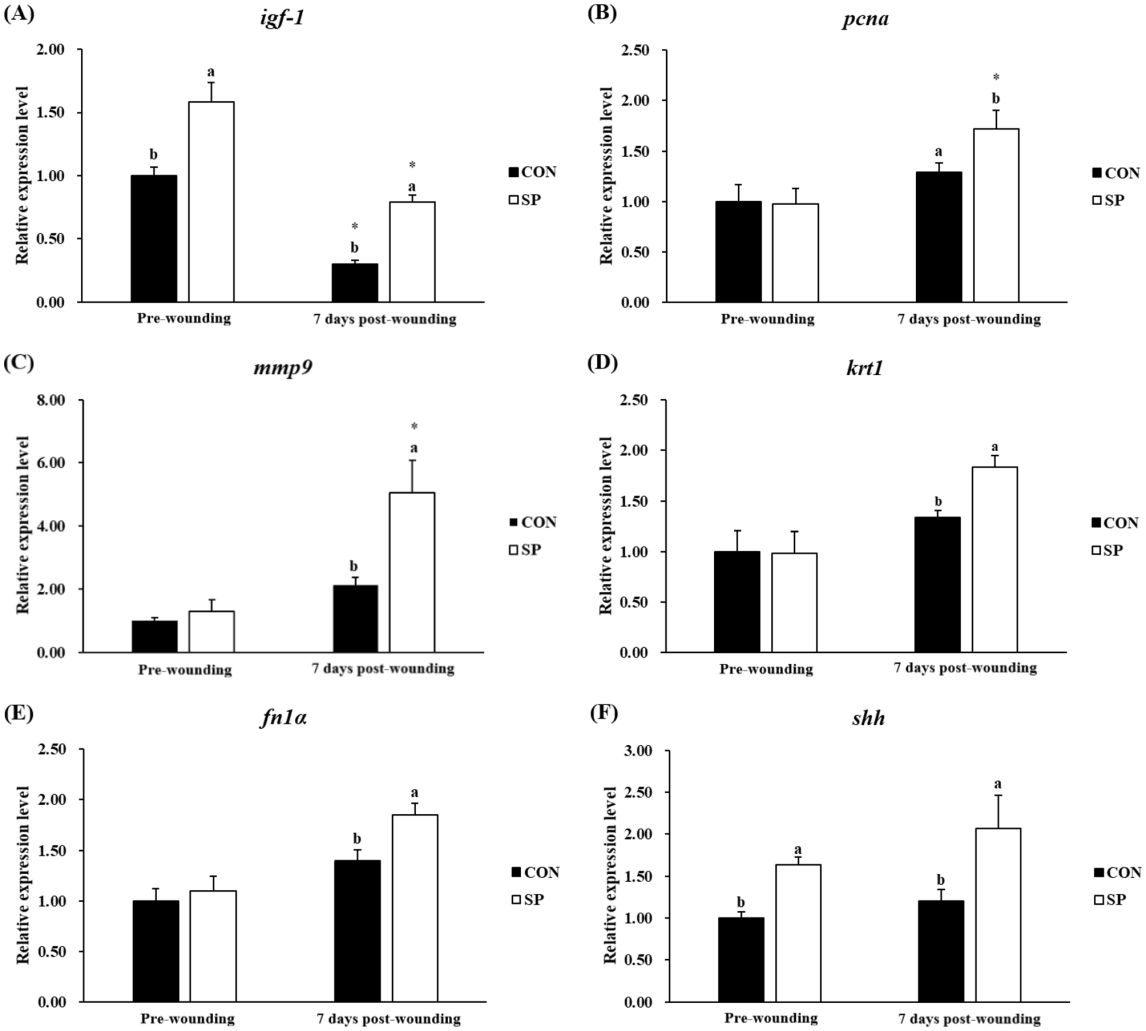
Relative expression level of genes involved in wound healing (*igf-1*, *pcna*, *mmp9*, *krt1*, *fn1α* and *shh*) in skin samples of gilthead seabream fed CON (control, black bars) or SP (control diet enriched with 10^9^ cfu g^−1^ SpPdp11, white bars) diet sampled pre-wounding or 7 days post-wounding. Error bars of columns denote standard error of means (n = 8). Different letters denoted significant differences between diets and asterisks indicated differences between time points (*P* < 0.05).

**Figure 5:**
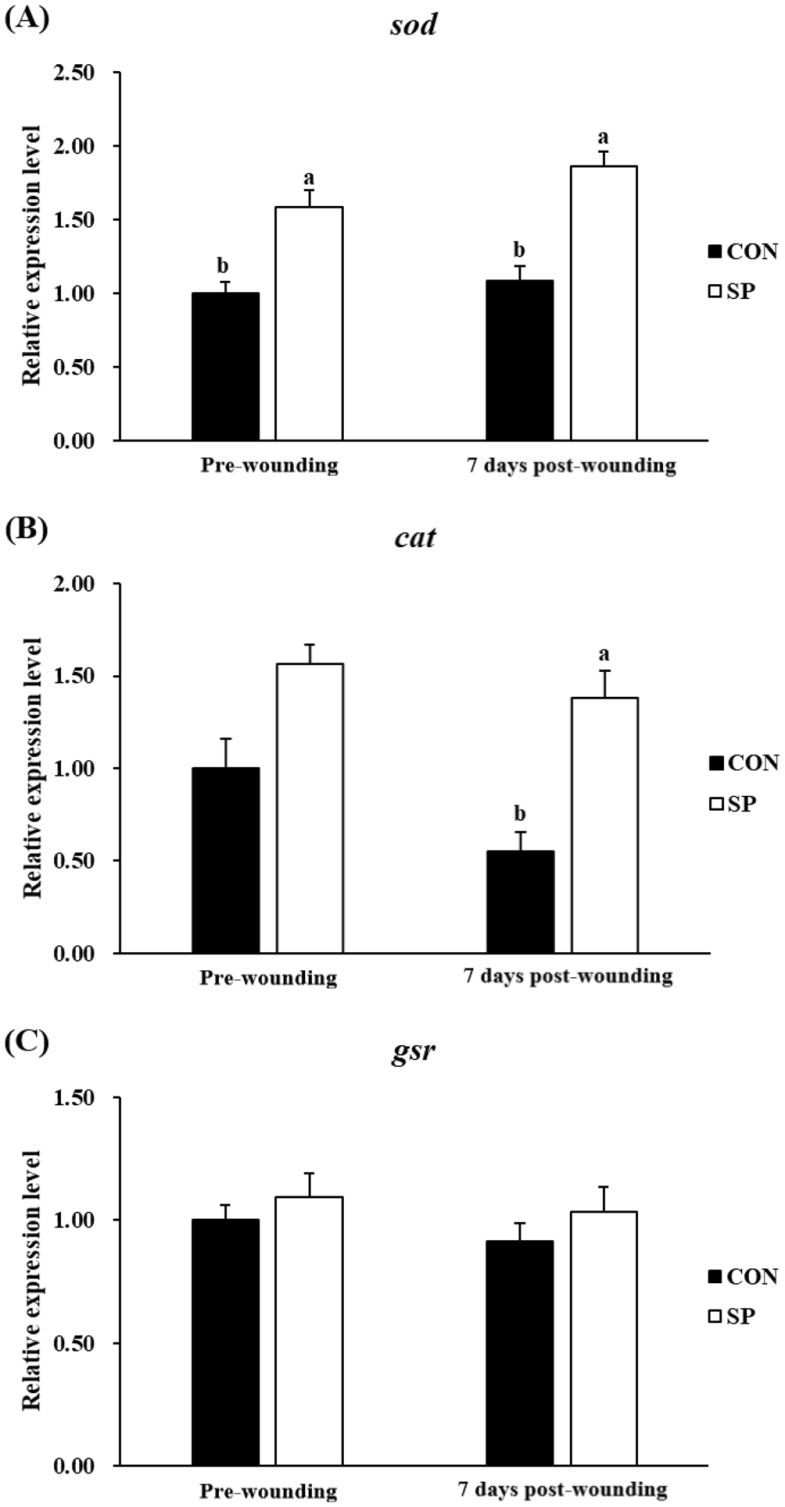
Relative expression level of antioxidant enzymes *sod*, *cat* and *gsr* in skin samples of gilthead seabream fed CON (control, black bars) or SP (control diet enriched with 10^9^ cfu g^−1^ SpPdp11, white bars) diet sampled pre-wounding or 7 days post-wounding. Error bars of columns denote standard error of means (n = 8). Different letters denoted significant differences between diets (*P* < 0.05).

The expression profile of fifteen genes was tested by real-time PCR in the skin of gilthead seabream from the different experimental groups: six pro-inflammatory or anti-inflammatory cytokines (*il-1β*, *il-6*, *il-8*, *tnf-α*, *il-10* and *tgf-β1*) (Fig. 3), six genes involved in wound healing (*igf-1*, *pcna*, *mmp9*, *krt1*, *fn1α* and *shh*) (Fig. 4), and three antioxidant enzymes (*sod*, *cat* and *gsr*) (Fig. 5).

### Inflammation

Regarding gene expression of genes related to inflammation, in fish pre-wounding, only *il-10* gene expression was significantly (*P* < 0.05) up-regulated in fish fed SP diet, compared with the CON group, while no significant (*P* > 0.05) changes were detected for the other studied genes (Fig. 3).

In the CON group, the skin wounds significantly (*P* < 0.05) enhanced the gene expression of *il-1β* and *il-8*, significantly (*P* < 0.05) suppressed the gene expression of *tgf-β1*, while no significant (*P* > 0.05) changes were detected for the other studied genes (Fig. 3). There were no significant (*P* > 0.05) changes induced by the skin wounds in the SP group (Fig. 3).

On the other hand, the expression of *il-1β* and *il-8* were significantly (*P* < 0.05) decreased, while the expression of *il-10*, and *tgf-β1* were significantly (*P* < 0.05) increased in SP group 7 days post-wounding by comparison with the CON group 7 days post-wounding (Fig. 3). No statistically significant (*P* > 0.05) variations were recorded for the gene expression of *il-6* and *tnf-α* gene expression among the different experimental groups 7 days post-wounding (Fig. 3).

### Re-epithelialization and ECM biosynthesis

Pre-wounding gilthead seabream fed SP diet displayed a significantly (*P* < 0.05) increased gene expression of *igf-1* and *shh*, as well as a no significantly (*P* > 0.05) changed gene expression of *pcna*, *mmp9*, *krt1*, *fn1α* compared with the fish in the CON group pre-wounding (Fig. 4).

There was a significant (*P* < 0.05) reduction in the *igf-1* expression but no significant (*P* > 0.05) change in the *pcna*, *mmp9*, *krt1*, *fn1α* and *shh* expression in the CON group between the fish pre-wounding and the fish 7 days post-wounding (Fig. 4). As for the SP group, the skin wound significantly (*P* < 0.05) suppressed the gene expression of *igf-1*, significantly (*P* < 0.05) enhanced the gene expression of *mmp9* and *pcna*, but not significantly (*P* > 0.05) changed the gene expression of *krt1*, *fn1α*, *and shh* (Fig. 4).

Furthermore, all the studied genes (*igf-1*, *pcna*, *mmp9*, *fn1α*, *krt1* and *shh*) were statistically significant (*P* < 0.05) up-regulated in fish fed SP diet, compared with those of CON group, when sampling 7 days post-wounding (Fig. 4).

### Antioxidant enzymes

Finally, no significant (*P* > 0.05) differences in the gene expression of *sod*, *cat*, and *gsr* between the fish pre-wounding and the fish 7-days post-wounding in both CON group and SP group were detected (Fig. 5). The expression of *sod* was significantly (*P* < 0.05) increased in fish fed SP diet, comparing to the values obtained in fish fed CON diet pre-wounding and 7 days post-wounding (Fig. 5). There was no significant (*P* > 0.05) differences in the gene expression of *gsr* in the SP group compared with the CON group, both pre-wounding and 7 days post-wounding (Fig. 5). Only statistically significant (*P* < 0.05) increases of the *cat* gene expression were observed in fish fed SP diet 7 days post-wounding but not (*P* > 0.05) pre-wounding compared with the fish in the CON group (Fig. 5).

## Discussion

The present study mainly showed that dietary administration of SpPdp11 at a dosage of 10^9^ cfu g^−1^ causes an appreciable positive effect on the skin wound healing of experimentally wounded gilthead seabream specimens. This fact was firstly demonstrated by the decrease of the wound areas detected macroscopically and confirmed by the image analysis, in those fish fed the probiotic diet. Furthermore, elevated presence of immune-related enzymes in the skin mucus of those fish was also corroborated, while alleviated inflammatory response through intensifying the antioxidant system, enhanced re-epithelialization, neo-vascularization and synthesis of matrix proteins in the skin, tested by the real-time PCR, was also encountered in fish fed SP diet, in comparison with fish fed the control diet. In a previous paper, we demonstrated that experimental wounds made on the left flank of gilthead seabream, in the central part of the body and below the lateral line were almost closed after 15 days^27^. Taken these results into account, we selected to study the healing process in the present work 7 days post-wounding.

The serum metabolic parameters have been widely used to reflect any modifications in the vital organs of organisms, under the action of some perturbing factors^28^. Skin lesions, abrasions or ulcers are known to cause changes in serum physiological and biochemical parameters, resulting in disorders of the stress response and systemic immune response in fish and higher vertebrate^29–32^. Nine different parameters (AST, CK, UA, GLU, Ca^2+^, PHOS, ALB/GLOB, K^+^, and Na^+^) were studied in the serum of gilthead seabream to investigate the possible alterations in the serum content induced by the skin wound and/or by the effect of the dietary administration of the probiotic SpPdp11. The skin wounds significantly disturbed the serum AST, UA, GLU and PHOS concentration and ALB/GLOB ratio. The levels of AST and ALB/GLOB were modified as results of the dietary administration of SP diet and/or the experimental wounds carried out on some fish. AST, which is very abundant in most of the tissues, is elevated in tissue necrosis during tissue degeneration^33,34^. AST is released from the cells into the blood, which is reflected by the increased AST activity in the serum^35^. The studies carried out on channel catfish (*Ictalurus punctatus*) and burn-induced Wistar albino rats, in which external injuries were studied, also led to an enhanced AST activity in serum^31,32^. Similarly, we found that the experimental wounds done on gilthead seabream led increments in AST activity in serum of fish fed CON diet sampled 7 days post-wounding. However, the activity of AST was not increased in fish sampled at that point but fed with the probiotic-treated diet (SP diet). This seems to indicate better tissue integrity in these wounded fish, as it was also verified by the data of the wound areas.

The ALB/GLOB ratio reflects the relative changes in the serum content^36^. Particularly, the albumin has numerous important functions in addition to its primary function of maintaining the plasma oncotic pressure, including ligand binding, antioxidant properties, and scavenging reactive oxygen and nitrogen species^37^. On the other hand, the globulin in serum is secreted from immune cells, and it is an important component to fight infections in animals, including fish^38^. It has been described that the increased globulin level in serum induced by severe skin lesions could result in a lower ALB/GLOB^39,40^, indicating a disease status with significant physiological implications on the immune capacity^41,42^. In agreement with those findings, in the present study, the skin wounds significantly reduced the ALB/GLOB ratio. However, SP diet enhanced the ALB/GLOB in fish sampled 7 days post-wounding, reflecting a better immune status in the SP group by comparison with the CON group.

Re-epithelialization is the term used to describe the resurfacing of a skin wound with new epithelium, which relies on the migration and proliferation keratinocytes^10,43,44^. There is an in vitro study showing that lysates of probiotics *L. rhamnosus* GG and *L. reuteri* significantly increased proliferation and migration in primary human keratinocyte monolayers^45^. In the present study, dietary SpPdp11 inclusion also seemed to indicate a higher keratinocytes migration and proliferation on the periphery of gilthead seabream skin wounds, when compared with the fish fed the non-supplemented diet (control diet), as demonstrated by the areas of wounds 7 days post-wounding (already discussed), but also by the gene expression of the genes selected for the present study (explained below).

Re-epithelialization is executed and regulated by an equally complex signaling network involving numerous growth factors. Of importance is the transforming growth factor β family and insulin-like growth factor family^46,47^. During the healing process in the proliferation phase, the increased number of cells in the wound bed relates to the release of many growth factors. In the present study, the gene expression of *igf-1* and *tgf-β1* in the SP group was significantly enhanced compared to the values of the CON group. Furthermore, another indication of the keratinocyte movement and adhesion was the up-regulation of *krt1* and *pcna* genes found also in fish from SP group sampled 7 days post-wounding. As a matter of fact, the synthesis of keratins, turned on by the migrating keratinocytes, are speculated to been linked to the migration of keratinocytes because of its facilitation in cell adhesion^48,49^, while the expression of *pcna* reflects the proliferation of epithelial cells, because this gene is essential for DNA replication^50^.

On the other hand, proteolytic degradation of extracellular matrix (ECM) bymatrix metalloproteinases (*mmps*) is also involved in the re-epithelialization^51^. As an important subtype of proteases^52^, *mmps* produced by migrating keratinocytes, facilitate the re-epithelialization by degrading dermal–epidermal junction components, releasing growth factors from the wound matrix and converting these growth factors to their active forms^43^. In the present study, consistent with the enhanced activity of proteases activity in skin mucus, the expression of *mmp9* was significantly escalated in the SP group. Dietary administration of SPdp11 may facilitate the removal of necrotic tissues and migration and proliferation of keratinocytes through increasing the synthesis of *mmps*, which is in favor of the re-epithelialization. Different from the previous study that the re-epithelialization of the epidermis continued for only 2 days in gilthead seabream skin after scale removal^7^, the progress of re-epithelialization continued after 7 days of recovering in the present study. This difference may result from that the healing of full-thickness excised skin lesions creates accompanies with a longer duration than that of the incision.

Reactive oxygen species (ROS) are essential secondary messenger signaling molecules for many immune cells and appear to be important in coordinating the recruitment of immune cells to the wound site and effective tissue repair. The oxidative stress posed by continuous ROS production regulated the recruitment of immune cells and induced the sustained pro-inflammatory cytokine secretion, which leads the DNA damage and cell apoptosis thereby affected the healing up of the wound^53^. Cellular protection against excessive oxidation is achieved mainly by enzymes, known members are as *sod*, *cat*, and *gsr*^54^. In accordance with our previous studies on gilthead seabream that dietary inclusion of SpPdp11 significantly enhanced the activities of antioxidants in head-kidney leucocytes, serum and skin mucus^19,20,22^, present results showed that SpPdp11 inclusion significantly enhanced the activity of peroxidase in mucus and the gene expression of *sod* and *cat* in the skin, showing the enhancement of antioxidant defenses. The excessive ROS not scavenged by the innate antioxidant system could trigger the signaling pathways of cytokine release. Previous studies in gilthead seabream indicated that the pathogenesis of the wound healing was mediated by the release of inflammatory cytokines^9^. The gene expression of *il-1β*, *il-6*, *il-8*, *tnf-α*, *il-10*, and *tgf-β1* was tested in the present study. Regarding two pro-inflammatory cytokines (*il-1β* and *il-8*), the related expression in the skin were significantly decreased in the group fed SP diet, compared to the control group. However, the expression of *il-10* and *tgf-β1*, which performing anti-inflammatory effects in most of the cases, was significantly up-regulated in the SP group. These results are in agreement with a previous in vitro study carried out in our laboratory, in which it was demonstrated that SpPdp11 was able to ameliorate the effects of a pathogenic *Vibrio* in the ventral skin of gilthead seabream^21^. Present results showed that dietary administration of SpPdp11 could mitigate the wound-induced skin inflammatory process by suppressing the pro-inflammatory cytokines and inducing the anti-inflammatory cytokines. Inflammation prepares the wound for the subsequent phases of healing and could be divided into an early phase and a late phase^55^. Pro-inflammatory cytokines over-express after wounding and trigger the synthesis of *mmps*, as well as inhibit the reconstruction of ECM during the early stage of the inflammatory phase^51^. As our previous study on the experimentally wounded gilthead seabream shown, the size of the wounded areas increased immediately after wounding and then start to fall as the day progresses^9^. As the image analysis results showed, the wound area was significantly smaller in SP group. So, in conclusion, dietary administration of SpPdp11 may narrow the early stage of the inflammatory phase, facilitate the skin wound healing.

ECM biosynthesis, the process that collagen matrix replacing the temporary matrix formed mainly from fibrin and fibronectin network in and further lead to restoring the structure and function of the proper tissue, is intensified after the inflammatory phase^10^. The fibroblast migration induced by the cytokines and growth factors has the main effect in the synthesis of ECM components^47,56^, which contributes to the granulation tissue formation^57,58^. Abound in the granulation tissue, fibronectin (*fn*) cooperates with hyaluronic acid to formulate the early granulation tissue^59^, which acts as a scaffold for new collagen deposition^12^. In our study, the *fn1α* was up-regulated in SP group by comparison with the CON group in fish sampled 7 days post-wounding, which meant that the addition of SpPdp11 promoted the formation of the early granulation tissue. Nevertheless, in the study on gilthead seabream, the early granulation tissue constituted with the *fn* acted as a substratum for the collagen only by the second day after scale removal, and then the collagen played the leading role^7^. These differences may be due to the different wound types between these two studies. Finally, angiogenesis is a crucial process in the normal wound healing, involving the neo-vascularizationin the wound site, which prevents the development of ischemic necrosis simultaneously stimulating the tissue repair process^10,60^. Sonic hedgehog (*shh*) signal pathway widely participates in regulating the development of skin, exhibits improvements in vascularity by increasing the recruitment of endothelial progenitor cells to wound vasculature^61^.

On the other hand, the results on diabetic mice showed that *shh* treatment could intensify the collagen assembly so as to promote ECM protein deposition^62^. Our results revealed that the expression of *shh* was significantly enhanced by the administration of SpPdp11, which indicated that this probiotic could help the neovascularization and synthesis of matrix proteins in the wound site. However, further studies are needed to know how the probiotic is involved in such molecular and cellular processes.

To conclude, the results presented in this study suggested that dietary SpPdp11 administration improved the skin repair in gilthead seabream by facilitating the wound closure and the re-epithelialization of the wounds. Furthermore, the probiotic alleviated the inflammatory response caused by the wounds by the homeostatic control of cellular ROS levels, promoted the vascularization and ECM protein deposition of the wound area. The precise mechanisms through which SpPdp11 facilitated the skin repair of experimentally wounded gilthead seabream need to be further elucidated by future studies.

## Materials and methods

### Ethics statement

The experiment was conducted at the Marine Fish Facilities at the University of Murcia (Spain). All experimental protocols were approved by the Ethical Committee of the University of Murcia (Permit Number CEEA 357/2017) following the guidelines of European Union for animal handling (2010/63/EU).

### Bacterial growth and maintenance

SpPdp11 strain was isolated from the skin of healthy specimens of *S. aurata*^63^, and grown in tryptone soya broth (OXOID Ltd., Basingstoke, UK) supplemented with 1.5% NaCl (TSBs, 20 h, 20 °C) with continuous shaking. Bacteria recovered from the plates were washed in sterile phosphate-buffered saline (PBS, pH 7.4) with continuous shaking. The density of bacterial suspension was determined by using a Z2 Coulter Particle Counter (BECKMAN Coulter, Barcelona, Spain), and adjusted to the required volume of bacterial solution was calculated according to the target additive (10^9^ cfu g^−1^).

### Diets and fish husbandry

A total of 48 specimens of gilthead seabream were purchased from a local farm (San Pedro del Pinatar, Murcia, Spain) with a mean initial body weight of 21.81 g (0.87 g SEM). Fish were acclimated for 2 weeks and fed a commercial pellet diet (Skretting) at a rate of 2% body weight per day. Then, they were randomly sorted into 4 re-circulating seawater aquaria (400 L), with continuous aeration and flow-through seawater at the rate of 900 L h^−1^ at 22 ± 2 °C and 24% salinity. An artificial photoperiod of 12 h light: 12 h dark was established. A commercial diet was used as the basic feed. Phosphate saline buffer (PBS) or bacterial suspension of equal volumes were added to the basic feed, formulating the CON (PBS) and SP (10^9^ cfu g^−1^) diets. Two tanks of 12 fish (two replicates) were fed each one of the two experimental diets at a rate of 2% body weight day^−1^. The specific feeding dosage was changed at any time according to the weight of the fish. Fish were fed twice a day at 7 a.m and 7 p.m^9,19^.

### Sample collection and wounds

After 30 days of the feeding trial, four non-wounded fish per tank were selected at random for sampling. Fish were anesthetized with 20 mg L^−1^ of clove oil (GUINAMA). Mucus for the immune parameter analysis was gently collected with a cell scraper (SIGMA-ALDRICH) from the whole-body skin surface of fish, taking care in avoiding any possible contamination with urinogenital and/or intestinal excretions. The supernatants of skin mucus were taken after centrifuged (1,400 g, 10 min, 4 °C) and then stored at − 20 °C until use. Serum was collected after centrifugation (4,000 g, 10 min) and stored at − 80 °C as separate aliquots until analysis. For gene expression evaluation, skin samples taken from the intact skin from the same place where the wound was made in wounded fish (explained below) were collected frozen in liquid nitrogen immediately and stored at − 80 °C pending analysis.

Four fish in each tank were sedated as previously mentioned and then wounded with a metallic circular biopsy punch (STIEKEL) with a diameter of 8 mm and 2 mm depth below the lateral line in the middle part of the left flank. Afterward, fish returned to the anesthetic-free water tank and recovered from anesthesia (usually this process took 5 min). Next, fish returned to their tank for continuing to be feed with the same experimental diet (CON or SP) for an extra week (7 days). At the end of this experimental time (7 days post-wounding), these fish were sampled as previously described for obtaining skin mucus, serum and skin samples (taken from the edge of the wound) for making the same analysis that those developed in fish sampled after 30 days of trial (pre-wounding).

### Image analysis of wound areas

For macroscopic observation of the healing process, just after making the wounds and at the end of the experiment, images of the wounds were taken. The images were analyzed by an image analysis software, IMAGE-PRO Plus version 6 (MEDIA CIBERNETICS, Silver Spring, MD, USA). The edge of the skin wound was traced by the brush and the wound area was selected. The selected area was measured automatically after activating spatial calibration shown in the background. Differences in the area were calculated by IBM SPSS Statistics for WINDOWS Version 22.0.

### Determination of metabolic parameters in serum

Quantitative determination of aspartate aminotransferase (AST), bile acids (BA), creatine kinase (CK), uric acid (UA), glucose (GLU), total calcium (CA^++^), phosphorus (PHOS), total protein (TP), albumin (ALB), globulin (GLOB), potassium (K^+^), sodium (Na^+^) in fish serum was determined by using Abaxis Veterinary Diagnostics (VETSCAN) equipped with Avian-Reptilian Profile Plus rotor (VETSCAN) according to the manufacturer's instructions.

### Protease activity in skin mucus

Protease activity was quantified using the azocasein hydrolysis assay according to the previous method^64^. Briefly, the positive control (100 µl of 5 mg ml^−1^ trypsin), the negative control (100 µl of 100 mM ammonium bicarbonate buffer), and the test samples (100 µl of skin mucus) were mixed with 100 µl of 100 mM ammonium bicarbonate buffer (pH = 8.6) containing 0.7% azocasein (SIGMA-ALDRICH), following 250 µl of 4.6% trichloroacetic acid (TCA) addition after incubation at RT for 24 h. The mixtures were centrifuged (6,000 g, 5 min), and the supernatants collected into a 96 wells microplate were read at 450 nm in a plate reader (FLUOSTAR OMEGA, BMG). The trypsin activity (%) = Samples optical density (OD) * (Positive control OD – Negative control OD)^–1 ^* 100.

### Anti-protease activity in skin mucus

The total anti-protease activity was defined as the capacity of trypsin inhibition^65^. The positive control (20 µL of ammonium bicarbonate buffer), the negative control (10 µl of 100 mM ammonium bicarbonate buffer + 10 µL of 5 mg mL^−1^ trypsin), and the test samples (10 µL of skin mucus samples + 10 µL 5 mg mL^−1^ trypsin) were respectively were incubated at room temperature for 10 min. And then, all the samples were mixed with 100 µL of 100 mM ammonium bicarbonate buffer and 125 µL of 0.7% azocasein (SIGMA). The reaction was stopped by adding 250 µL of 4.6% TCA after incubation for 2 h at room temperature, and the mixture was centrifuged (6,000×*g*, 5 min). The OD of supernatants was read at 450 nm in a plate reader (FLUOSTAR OMEGA, BMG). The antiprotease activity (%) = 100 – Samples OD * (Negative control OD – Positive control OD)^–1 ^* 100.

### Peroxidase activity in skin mucus

The peroxidase activity was determined by a colorimetric method as described by^66^. Briefly, aliquots of 10 μL of skin mucus were diluted with 40 μL of Hank’s buffer (HBSS) without Ca^2+^ or Mg^2+^ in flat-bottomed 96-well plates. 100 μL of 10 mM TMB and 0.015% H_2_O_2_were added per well and used as the substrates of the reaction. The color-change reaction was stopped after 2 min by adding 50 μL of 2 M sulphuric acid (H_2_SO_4_) and the OD was read at 450 nm in a plate reader. Standard samples without skin mucus were used as blanks. The enzyme activity is expressed as specific activity, normalized by the tissue protein. The protein concentration of skin mucus homogenates was determined using the Bradford dye-binding kit (SIGMA).

### RNA extraction and qPCR

The total RNA of the skin was isolated using Trizol reagent (LIFE TECHNOLOGIES) and then homogenized with a handheld tissue homogenizer (AVANS). RNA purity and concentration were measured using Nano Drop 2000 spectrophotometer (THERMO FISHER SCIENTIFIC) and then treated with DNase I (PROMEGA) to remove genomic DNA contamination. First-strand complementary DNA (cDNA) synthesis was reversely transcribed from 1 μg of total RNA using the SuperScript IV reverse transcriptase (LIFE TECHNOLOGIES) with an oligo-dT18 primer (LIFE TECHNOLOGIES). Six pro-inflammatory or anti-inflammatory cytokines (*il-1β*, *il-6*, *il-8*, *tnf-α*, *il-10* and *tgf-β1*), six genes involved in wound healing (*igf-1*, *pcna*, *mmp9*, *krt1*, *fn1α* and *shh*), and three antioxidant enzymes (*sod*, *cat* and *gsr*) were analyzed. Specific primers for target genes and housekeeping genes were listed in Table 2. All the real-time PCR analyses were performed using an ABIPRISM 7,500 Instrument (APPLIED BIOSYSTEMS) with using SYBR Green PCR Core Reagents (APPLIED BIOSYSTEMS). The qRT-PCR conditions were: 95 °C for 10 min and then 40 cycles of 95 °C for 15 s, 58 °C for 15 s, and 72 °C for 40 s. The mRNA levels of these genes were normalized to the mRNA level of sea bream *ef1a* and the *rps18*, which were used as housekeeping genes. The gene expression levels were calculated using the 2^–ΔΔCT^ method^67^, and the relative expression level of the gene in fish of CON group pre-wounding was used as a calibrator.

**Table 2:** Primers used for real-time PCR analysis.

Target gene	Forward primer (5′–3′)	Reverse primer (5′–3′)	GenBank no
*ef1α*	TGTCATCAAGGCTGTTGAGC	GCACACTTCTTGTTGCTGGA	AF184170
*rps18*	CGAAAGCATTTGCCAAGAAT	AGTTGGCACCGTTTATGGTC	AM490061
*il-1β*	GGGCTGAACAACAGCACTCTC	TTAACACTCTCCACCCTCCA	AJ277166
*il-6*	AGGCAGGAGTTTGAAGCTGA	ATGCTGAAGTTGGTGGAAGG	AM749958
*il-8*	GCCACTCTGAAGAGGACAGG	TTTGGTTGTCTTTGGTCGAA	AM765841
*tnf-α*	TCGTTCAGAGTCTCCTGCAG	TCGCGCTACTCAGAGTCCATG	AJ413189
*il-10*	CTCACATGCAGTCCATCCAG	TGTGATGTCAAACGGTTGCT	FG261948
*tgf-β1*	GCATGTGGCAGAGATGAAGA	TTCAGCATGATACGGCAGAG	AF424703
*igf-1*	AGGACAGCACAGCAGCCAGACAAGAC	TTCGGACCATTGTTAGCCTCCTCTCTG	AY996779
*pcna*	GAGCAGCTGGGTATTCCAGA	CTGTGGCGGAGAACTTGACT	P12004
*mmp9*	ATTCAGAAGGTGGAGGGAGCG	CATTGGGGACACCACCGAAGA	AM905938
*krt1*	AGAGATCAATGACCTGCGGC	CCCTCTGTGTCTGCCAATGT	FJ744592
*fn1α*	CGGTAATAACTACAGAATCGGTGAG	CGCATTTGAACTCGCCCTTG	FG262933
*shh*	TGTCCTTGGTGTCCTCTGGGATGGG	GGAGTTTCTTGTGATCTTGCCTTC	FM153740
*sod*	CCATGGTAAGAATCATGGCGG	CGTGGATCACCATGGTTCTG	AJ937872
*cat*	TTCCCGTCCTTCATTCACTC	CTCCAGAAGTCCCACACCAT	FG264808
*gsr*	CAAAGCGCAGTGTGATTGTGG	CCACTCCGGAGTTTTGCATTTC	AJ937873

*ef1α* elongation factor 1α, *rps18* ribosomal proteinS18, *il-1β* interleukin-1β, *il-6* interleukin-6, *il-8* interleukin-8, *tnf-α* tumor necrosis factor-α, *il-10* interleukin-10, *tgf-β1* transforming growth factor-β1, *igf-1* insulin-like growth factor 1, *pcna* proliferating cell nuclear antigen, *mmp9* matrix metalloproteinase 9, *krt1* keratin type 1, *fn1α* fibronectin 1α, *shh* sonic-hedgehog, *sod* Cu Zn-superoxidedismutase, *cat* catalase, *gsr* glutathione reductase.

### Statistical analysis

Data were statistically analyzed by two-way ANOVA using IBM SPSS Statistics for WINDOWS Version 22.0 for WINDOWS. Differences between the two groups in each time point and differences between two time points in each group were tested. Differences were regarded as significant when *P* < 0.05 and the results are presented as means ± SEM (standard error of the mean). Different letters denoted significant differences between diets and asterisks indicated differences between time points.
